# HIV risk and prevention among clients of a delivery-based harm reduction service during an HIV outbreak among people who use drugs in northern rural Minnesota, USA

**DOI:** 10.1186/s12954-023-00839-1

**Published:** 2023-08-02

**Authors:** Adam Palayew, Kelly Knudtson, Sue Purchase, Shayna Clark, Lucia Possehl, Elise Healy, Sarah Deutsch, Courtney A. McKnight, Don Des Jarlais, Sara N. Glick

**Affiliations:** 1 Department of Epidemiology, University of Washington, Seattle, WA USA; 2 Division of Allergy and Infectious Diseases, Department of Medicine, University of Washington, WA Seattle, USA; 3 Harm Reduction Sisters, Duluth, MN USA; 4 School of Global Public Health, New York University, New York City, NY USA

**Keywords:** Syringe service programs, HIV, Minnesota, People who inject drugs

## Abstract

**Background:**

Since 2019, multiple HIV outbreaks occurred among people who inject drugs (PWID) in Minnesota. Syringe service programs (SSPs) are evidence-based programs that reduce the spread of HIV. We conducted an assessment of characteristics and HIV risk and prevention among clients of a delivery-based SSP near an HIV outbreak in rural, northern Minnesota.

**Methods:**

In the fall of 2021, we conducted a cross-sectional survey of clients of a mobile SSP based in Duluth, Minnesota. Survey topics included demographics, drug use, sexual behavior, HIV testing history, and HIV status. We conducted descriptive analyses and used univariate logistic regression to identify correlates of syringe sharing. The analysis was limited to PWID in the last six months.

**Results:**

A total of 125 people were surveyed; 77 (62%) were PWID in the last six months. Among these participants, 52% were female and 50% were homeless. Thirty-two percent reported sharing syringes and 45% reported sharing injecting equipment. Approximately one-half (49%) of participants had been tested for HIV in the past year, and none reported being HIV-positive. Individuals reported low condom usage (88% never used), and 23% of participants reported engaging in some form of transactional sex in the last six months. Incarceration in the last year was associated with sharing syringes (odds ratio = 1.4, 95% confidence interval 1.1–1.8).

**Conclusion:**

HIV risk was high among PWID receiving services at this SSP. These data highlight a rural SSP that is engaged with people at risk for HIV and needs additional support to expand harm reduction services.

**Supplementary Information:**

The online version contains supplementary material available at 10.1186/s12954-023-00839-1.

## Background

HIV disproportionately affects people who inject drugs (PWID). In the United States (US), there were approximately 30,635 new cases of HIV in 2020, with 7% among PWID [1]. Syringe service programs (SSPs) decrease HIV risk among PWID [2–6]. SSPs reduce the frequency of other outcomes such as incidence of hepatitis C virus (HCV), endocarditis, skin and soft tissue infections, and syringe litter [7–11].

In the past decade, there have been multiple HIV outbreaks among PWID in the US [12]. Minnesota has two ongoing HIV outbreaks. The outbreak in the Minneapolis/St. Paul area was identified in 2020 with cases dated back to 2018, and the Duluth outbreak was identified in 2021. Both of these outbreaks have disproportionately impacted the local Indigenous population [13]. The Minnesota Department of Health reported the outbreaks are among PWID, people experiencing homelessness, men who have sex with men, and people who engaged in transactional sex; there is evidence that the two outbreaks may be connected [14]. Previous to March 2020, Duluth had minimal services for PWID. The Duluth area has had an SSP operating since 2014 with a one-for-one exchange model. In May 2019, Harm Reduction Sisters (HRS), a needs-based SSP, began offering mobile, peer distribution, and mail-delivery syringe services across 13 counties, all considered rural by the US Census. In October 2021, HRS expanded services to provide HIV/HCV/syphilis testing, linkage to HIV care, and non-medical HIV case management. HRS has aimed to deliver services in rural areas by overcoming geographic and stigma-related barriers. This includes providing services as discretely as possible using mail- and delivery-based services. Additionally, by using a needs-based distribution model and peer distribution, HRS can provide supplies to people who are not directly accessing their services.

In this paper, we present the results of a 2021 collaboration between HRS and the University of Washington (UW) to conduct a survey to evaluate HRS participants’ HIV risk and prevention strategies, substance use patterns, and harm reduction needs in the context of an ongoing HIV outbreak.

## Methods

In fall 2021, HRS conducted a cross-sectional survey among their program clients. Data were collected between October 8, 2021–November 16, 2021, with the goal of evaluating services.

Clients were recruited during routine SSP services and at three pop-up HIV testing events on October 28th, November 12th, and November 16th, 2021. Eligibility criteria included all people receiving services, including people who do and do not use drugs. Sometimes staff were not able to accurately determine who engaged in SSP services.

Prior to data collection, interviewers obtained verbal consent. Data were collected by paper survey or REDCap; paper surveys were entered into REDCap [15]. The survey included 29 questions, was anonymous, and was administered by SSP staff. Questions included demographics, current needs, substance use, sexual behavior, and self-reported HIV testing and status. Due to an administrative error, 31 people did not receive the question about drugs consumed in the last six months. All participants who reported being HIV negative were eligible and offered HIV testing; those who tested completed a longer version of the survey (40 questions) prior to testing, with additional required HIV questions. All survey participants were compensated with a $20 Visa gift card for the survey regardless of testing.

This survey was conducted for program monitoring purposes and did not undergo IRB review. This specific analysis was determined to be non-human subjects research by the University of Washington’s Human Subjects Division.

### Data analysis

Because of the emphasis on HIV risk among PWID, we restricted these analyses to PWID in the last six months. Additional file 1 includes data from the full dataset stratified by injection drug use in the last six months to compare the demographics of those excluded.

Data were analyzed descriptively using R [16]. We calculated proportions for participants who provided complete data; participants who had missing data or refused to answer were excluded. We assessed the association between syringe sharing and pre-specified variables of interest using univariate logistic regression. Several variables were hypothesized a priori to be associated with syringe sharing, stigma of accessing needle exchange, and social marginalization and included: sex assigned at birth, housing status, incarceration in the past six months, and engaging in transactional sex in the last six months. Due to small sample size, we did not conduct any multivariable regression.

## Results

Overall, 125 individuals completed the survey; 77 (62%) reported injecting drugs in the last six months (Table 1). The median age was 36 years (interquartile range = 27–45). The majority (52%) were women. The two most commonly reported racial/ethnic identities were White (54%) and American Indian/Alaska Native (45%). Half of participants were homeless, 38% were unstably housed, and 12% had permanent housing. One-third reported being incarcerated in the last year. Individuals reported needing the most help with food (58%), wound care (20%), STI testing/treatment (11%), COVID support/testing (9%), and vaccinations (2%).

**Table 1:** Characteristics of participants who reported injection drug use in the past six months, SSP client survey, Northern Minnesota, 2021 (N = 77)

Characteristics	N = 77
**Age (median (Interquartile Range))**	36 (27–45)
Gender, n (column %)*^	
Man	34 (45)
Woman	39 (52)
Non-Binary	2 (3)
Total	75
**Sex at Birth, n (column %)**	
Female	39 (52)
Male	36 (48)
Total	75
Race/Ethnicity, n (column %)*	
American Indian/Alaska Native	33 (45)
Asian/South Asian	1 (1)
Black/African American	2 (3)
Native Hawaiian/Pacific Islander	1 (1)
White	40 (54)
Total	74
**Housing, n (column %)**	
Homeless	37 (50)
Temporary or unstable	28 (38)
Permanent	9 (12)
Total	74
**Incarcerated last year, n (column%)**	
Yes	19 (33)
No	38 (67)
Total	57
What do you need help with?, n (column%)*	
COVID-19/support and testing	5 (9)
Food support	32 (58)
STI testing/treatment	6 (11)
Wound care	11 (20)
Vaccinations	1 (2)
Total	55
**County, n (column %)**	
Carlton, MN	1 (1)
Koochiching, MN	3 (4)
St. Louis, MN	73 (95)
Total	77

^*^ Participants could select more than one response and declined to answer are excluded from denominator

^Other responses that could be selected were trans-male, trans-female, two-spirited, and other, but no one identified as any of these identities

Among PWID asked about drugs used, the most commonly used drugs in the last six months were methamphetamine alone (91%), heroin alone (54%), goofball (heroin + methamphetamine) (52%), fentanyl alone (43%), speedball (heroin + cocaine) (33%), and prescription opiates alone (24%). A full table of drugs used is in Additional file 2.

Among PWID in the past 6 months, 32% reported sharing syringes and 45% reported sharing other injection equipment (Table 2). Most participants (64%) never needed assistance with injection, while 11% always needed help, 13% needed help most of the time, and 13% needed help about half the time.

**Table 2:** Behavior related to HIV transmission risk and HIV in the last six months among participants who reported injection drug use in the past six months, SSP client survey, Northern Minnesota, 2021 (N = 77)

HIV Risk Behaviors	N = 77
Sharing syringes*	n, (%)
Yes	23 (32)
No	46 (64)
Unsure	3 (4)
Total	72
Sharing injecting equipment (excluding syringes)*	**n, (%)**
Yes	32 (45)
No	38 (54)
Unsure	1 (1)
Total	71
Assistance with injection*	**n, (%)**
Always	5 (11)
Most of the time (75–99%)	6 (13)
About half the time (26–74%)	6 (13)
Occasionally (1–25%)	0 (0)
Never	30 (64)
Total	47
Prior HIV test*	
Yes	51 (67)
No	24 (32)
I don't know	1 (1)
Total	76
*Result of last HIV Test^*	
Nonreactive/Negative	46 (94)
Unsure	3 (6)
Total	49 (4)
*Time of last HIV Test ^*	
2020-Present	25 (49)
2015–2019	15 (29)
2010–2014	4 (8)
Before 2010	2 (4)
Unsure	5 (10)
Total	51
Condom usage, last 6 months* +	
Always	2 (3)
Most of the time (75–99%)	0 (0)
About half the time (26–74%)	5 (8)
Occasionally (1–25%)	0 (0)
Never	52 (88)
Total	59
Engaged in transactional sex, last 6 months*	
Yes	16 (23)
No	54 (76)
Unsure	1 (1)
Total	71

^*^Decline to answer and missing were not included in the total

^Among those who ever received an HIV test as the denominator

+ In this only those who reported having a sex partner in the last six months were included in the denominator

Among PWID in the past six months, 67% had ever tested for HIV; of those who had ever tested, 94% were negative and 6% were unsure of their status (Table 2). HRS offered HIV testing to everyone who reported injecting drugs in the last 6 months with 16 agreeing. All HIV tests were negative.

Among PWID in the past six months, 65 (84%) reported having at least one sex partner in the last six months. Among these participants, 88% never used condoms, 3% always used condoms, and 8% used condoms half of the time (Table 2). Twenty-three percent of participants had engaged in some form of transactional sex in the last six months.

In the regression analysis, we found a statistically significant association between syringe sharing and incarceration in the past six months (odds ratio = 1.4, 95% CI 1.1–1.8). There were no significant associations between syringe sharing and sex assigned at birth, engaging in transactional sex in the last six months, or housing status.

## Discussion

Multiple recent HIV outbreaks among PWID reaffirm the critical role that SSPs provide in HIV prevention. As a small, rural SSP providing services in the midst of ongoing HIV outbreaks among PWID in Northern Minnesota, we undertook this survey to better understand the HIV risks and needs of SSP clients. HIV risk in this sample was high: 32% reported sharing syringes, approximately one-third had never been tested for HIV, 88% of sexually active clients reported never using condoms, and 23% had engaged in transactional sex in the last six months. The results reflect the complex competing needs of PWID. The majority of respondents reported being homeless and many reported unmet food and wound care needs. Additionally, a higher proportion of participants served by this SSP identified as female and Indigenous relative to other SSPs around the country [17, 18].

Syringe sharing is a risk factor for HIV transmission among PWID. We found that 32% of PWID also reported syringe sharing, which is similar to other published data among clients attending SSPs [19–21]. According to a 2022 study, rural areas have higher reported rates of syringe sharing on average relative to the proportion found in this study [20]. A high level of syringe sharing is concerning in the context of an ongoing HIV outbreak and low levels of HIV testing. In other places with HIV outbreaks, including Saskatoon, Saskatchewan, Canada, there has also been a lack of syringe coverage [12]. Moreover, the observed association between syringe sharing and recent incarceration indicates a notable gap in syringe coverage with a population vulnerable to HIV. These findings highlight the need for expanded syringe access in prisons as well as upon release to ensure sufficient syringe coverage.

Our survey also identified high levels of polysubstance use, including > 90% use of methamphetamine and > 40% use of fentanyl. High and increasing levels of methamphetamine use—both alone and in combination with heroin (known as goofball)—have been reported across the US [22, 23]. To our knowledge, this is the first report of near ubiquitous methamphetamine use among PWID in this area. Both methamphetamine and fentanyl use are associated with more frequent injection, and thus a need for higher levels of syringe distribution at SSPs [24, 25]. Additionally, there were high levels of reported condomless sex and transactional sex, adding another intersection of potential HIV transmission risk.

The uptake of HIV testing provided by HRS was modest with only 16 participants completing testing. Other research has also shown low levels of HIV testing among rural PWID [26]. Our survey did not ask participants about reasons for not receiving HIV testing, but prior studies have found barriers to HIV testing among rural PWID to be confidentiality concerns, negative treatment by healthcare workers, low perceived HIV risk, competing health priorities, and HIV related stigma [27–29]. Indeed, our survey did identify multiple unmet needs for HRS participants such as food, housing, and wound care. It is conceivable that participants with such high levels of basic needs are not in a position to prioritize HIV testing. This confluence of findings highlights the profound challenges that rural harm reduction services face. Providing these services in rural areas is particularly challenging due to large distances between services and where people live [30]. Moreover, because stigma related to substance use and harm reduction are particularly high in rural areas, SSPs often provide in-person, delivery-based services [31]. The logistics of delivery-based services may preclude the provision of more comprehensive support services [32]. Rural SSPs may also be less likely to have the funding, organizational capacity, and domain expertise of more urban SSPs, all of which can facilitated better connections to food assistance, housing assistance, and wound care [33].

This survey had limitations. During survey planning, we were not able to get approval to collect data on tribal lands and did not collect data on any local tribal lands. Native American clients who received services off tribal lands were included, but Native American clients who receive SSP services are likely underrepresented in this data. Second, due to an administrative error during survey implementation, not all survey participants were asked the question about recent drugs used which prevented more comprehensive analyses by specific drugs used. Third, due to high levels of missing data for some additional variables, some proportions should be interpreted with caution and may be underreporting the true prevalence, including incarceration history and assistance with injecting where missingness is likely to be non-random. Finally, there were difficulties navigating an incentivized survey with an SSP. At the public HIV testing pop-up events, staff had difficulty restricting the survey to only people who were receiving services, in part due to the $20 incentive gift card. Therefore, not all survey participants were previous clients and many were not PWID. We restricted our analysis to PWID both as an imperfect proxy of SSP clients and to focus on those most at risk for HIV.

## Conclusion

In conclusion, we found that the SSP was serving a population of PWID at elevated risk for HIV with a high proportion of women and Native American participants relative to other SSPs. Our findings indicate the need for further support and resources for SSPs, particularly those serving rural and marginalized populations. This need is not just for HIV prevention, but also for the multitude of other services needed by this community, including food access, housing options, wound care, and expanding harm reduction options for people using methamphetamine.

## Supplementary Information

**Additional file1**. **Supplemental Table 1:** Characteristics of all survey participants stratified by injection drug use in the last six months, SSP client survey, Northern Minnesota, 2021 (N=123).

**Additional file2**. **Supplementary Table 2.** Drug use among participants who reported injection drug use in the past six months, SSP client survey, Northern Minnesota, 2021 (N=46).

## Data Availability

The data that support the findings of this study are available from Harm Reduction Sisters but restrictions apply to the availability of these data, which were used under license for the current study, and so are not publicly available. Data are however available from the authors upon reasonable request and with permission of Harm Reduction Sisters.

## Abbreviations

PWID: People who inject drugs
SSP: Syringe service programs
HIV: Human immunodeficiency virus
US: United States
HCV: Hepatitis C virus
UW: University of Washington
COVID-19: Coronavirus disease 2019
OR: Odds ratio
CI: Confidence interval
