# Supplementary material for: HIV risk and prevention among clients of a delivery-based harm reduction service during an HIV outbreak among people who use drugs in northern rural Minnesota, USA
Source: Harm Reduct J. 2023 Aug 2;20:102. doi: 10.1186/s12954-023-00839-1 (PMC10394878; doi:10.1186/s12954-023-00839-1)
Supplement: Supplementary file 1 — Additional file1. Supplemental Table 1: Characteristics of all survey participants stratified by injection drug use in the last six months, SSP client survey, Northern Minnesota, 2021 (N=123). [file 12954_2023_839_MOESM1_ESM.docx]

**Supplemental Table 1**: Characteristics of all survey participants stratified by injection drug use in the last six months, SSP client survey, Northern Minnesota, 2021 (N=123)

|  | Injected drugs in the last six months | |
| --- | --- | --- |
|  | Yes (N=77)  n (%) | No (N=46)  n (%) |
| **Characteristics** |  |  |
| **Age (mean (standard deviation))** | 36 (9) | 42 (13) |
| **Gender*^** |  |  |
| Man | 34 (45) | 21 (48) |
| Woman | 39 (52) | 22 (50) |
| Non-Binary | 2 (3) | 1 (2) |
| Total | 75 | 44 |
| **Sex at Birth*** |  |  |
| Female | 39 (52) | 24 (52) |
| Male | 36 (48) | 22 (48) |
| Total | 75 | 46 |
| **Race/Ethnicity*** |  |  |
| American Indian/Alaska Native | 33 (45) | 22 (48) |
| Asian/South Asian | 1 (1) | 0 (0) |
| Black/African American | 2 (3) | 6 (13) |
| Native Hawaiian/Pacific Islander | 1 (1) | 1 (2) |
| White | 40 (54) | 25 (54) |
| Total | 74 | 46 |
| **Housing*** |  |  |
| Homeless | 37 (50) | 15 (34) |
| Temporary or unstable | 28 (38) | 8 (18) |
| Permanent | 9 (12) | 21 (48) |
| Total | 74 | 44 |
| **Incarcerated last year*** |  |  |
| Yes | 19 (33) | 6 (19) |
| No | 38 (67) | 25 (81) |
| Total | 57 | 31 |
| **What do you need help with?*** |  |  |
| COVID-19/support and testing | 5 (9) | 3 (11) |
| Food support | 32 (58) | 25 (89) |
| STI testing/treatment | 6 (11) | 3 (11) |
| Wound care | 11 (20) | 2 (7) |
| Vaccinations | 1 (2) | 1 (4) |
| Total | 55 | 28 |
| **County** |  |  |
| Carlton, MN | 1 (1) | 0 (0) |
| Koochiching, MN | 3 (4) | 3 (7) |
| St. Louis, MN | 73 (95) | 43 (94) |
| Total | 77 | 46 |

*Declined to answer and missing are excluded.

^Other responses included trans-male, trans-female, two-spirited, and other, but no one identified as any of these identities.
