# Supplementary material for: HIV risk and prevention among clients of a delivery-based harm reduction service during an HIV outbreak among people who use drugs in northern rural Minnesota, USA
Source: Harm Reduct J. 2023 Aug 2;20:102. doi: 10.1186/s12954-023-00839-1 (PMC10394878; doi:10.1186/s12954-023-00839-1)
Supplement: Supplementary file 2 — Additional file2. Supplementary Table 2. Drug use among participants who reported injection drug use in the past six months, SSP client survey, Northern Minnesota, 2021 (N=46). [file 12954_2023_839_MOESM2_ESM.docx]

**Supplementary Table 2**. Drug use among participants who reported injection drug use in the past six months, SSP client survey, Northern Minnesota, 2021 (N=46)

| **What drugs did you use in the last 6 months?*** | N=46  n (%) |
| --- | --- |
| Crack cocaine | 0 (0) |
| Fentanyl | 20 (43) |
| Heroin | 25 (54) |
| Methamphetamine | 42 (91) |
| Powder Cocaine | 10 (22) |
| Prescription Opiates | 11 (24) |
| Goofball (heroin/meth) | 24 (52) |
| Speedball (heroin/cocaine) | 15 (33) |
| Unsure | 2 (4) |
| Other | 1 (2) |

*Respondents could select more than one drug so the column sums to more than 100%.
